# Supplementary material for: Synergistic insights into human health from aptamer- and antibody-based proteomic profiling
Source: Nat Commun. 2021 Nov 24;12:6822. doi: 10.1038/s41467-021-27164-0 (PMC8613205; doi:10.1038/s41467-021-27164-0)
Supplement: Supplementary file 1 — Supplementary Information [file 41467_2021_27164_MOESM1_ESM.pdf]

SUPPLEMENTARY INFORMATION

Synergistic insights into human health from aptamer- and antibody-based proteomic profiling

Maik Pietzner[1,2], Eleanor Wheeler[1], Julia Carrasco-Zanini[1], Nicola D. Kerrison[1], Erin Oerton[1], Mine Koprulu[1], Jian'an Luan[1], Aroon D. Hingorani[3,4,5], Steve A. Williams[6], Nicholas J. Wareham[1,5], Claudia Langenberg[1,2,5]*

Affiliations

[1]MRC Epidemiology Unit, University of Cambridge, Cambridge, UK

[2]Computational Medicine, Berlin Institute of Health (BIH) at Charité – Universitätsmedizin Berlin, Germany

[3]Institute of Cardiovascular Science, Faculty of Population Health, University College London, London WC1E 6BT, UK

[4]UCL BHF Research Accelerator centre

[5]Health Data Research UK

[6]SomaLogic, Inc., Boulder, CO, USA

*Correspondence to Dr Claudia Langenberg (claudia.langenberg@mrc-epid.cam.ac.uk)

FIGURES

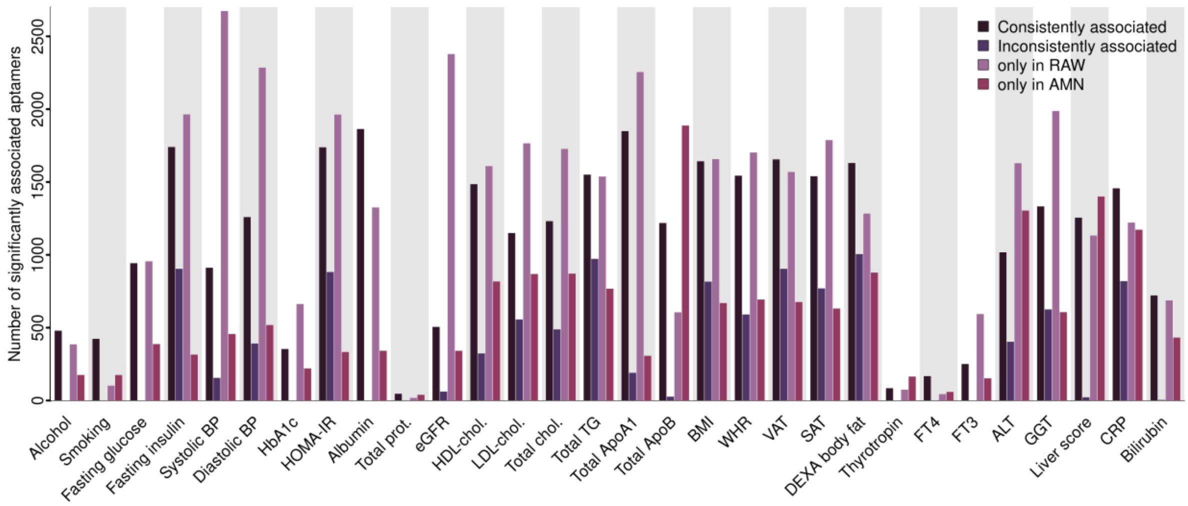

Supplementary Figure 1 Number of associated aptamers with a diverse set of phenotypic characteristics. For each exposure (x-axis), linear regression models were run associating all 4,979 aptamers covered on the SomaScan v4 platform as outcome adjusting for age and sex. This analysis was done twice using normalised (AMN) and non-normalised (RAW) data values for each aptamer. A stringent Bonferroni-correction was applied to declare statistical significance ($p<10^{-5}$). The bars depict 1) the number of consistently significantly associated aptamers across both data sets (dark purple, directionally concordant and significantly associated), 2) the number of aptamers significantly associated but with opposing effect directions (violet), 3) the number of aptamers significantly associated only when considering the non-normalised data (light purple), and 4) the number of aptamers significantly associated only when considering the normalised data. Source data are provided as a Source Data file. BP = blood pressure, HOMA-IR = homeostatic model of insulin resistance, prot. = protein, HDL-chol. = high-density lipoprotein cholesterol, LDL-chol. = Low-density lipoprotein cholesterol, TG = triglycerides, BMI = body mass index, VAT = visceral adipose tissue, SAT = subcutaneous adipose tissue, FT4 = free thyroxine, FT3 = free triiodothyronine, ALT = alanine aminotransferase, GGT = gamma-glutamyl transpeptidase, CRP = C-reactive protein

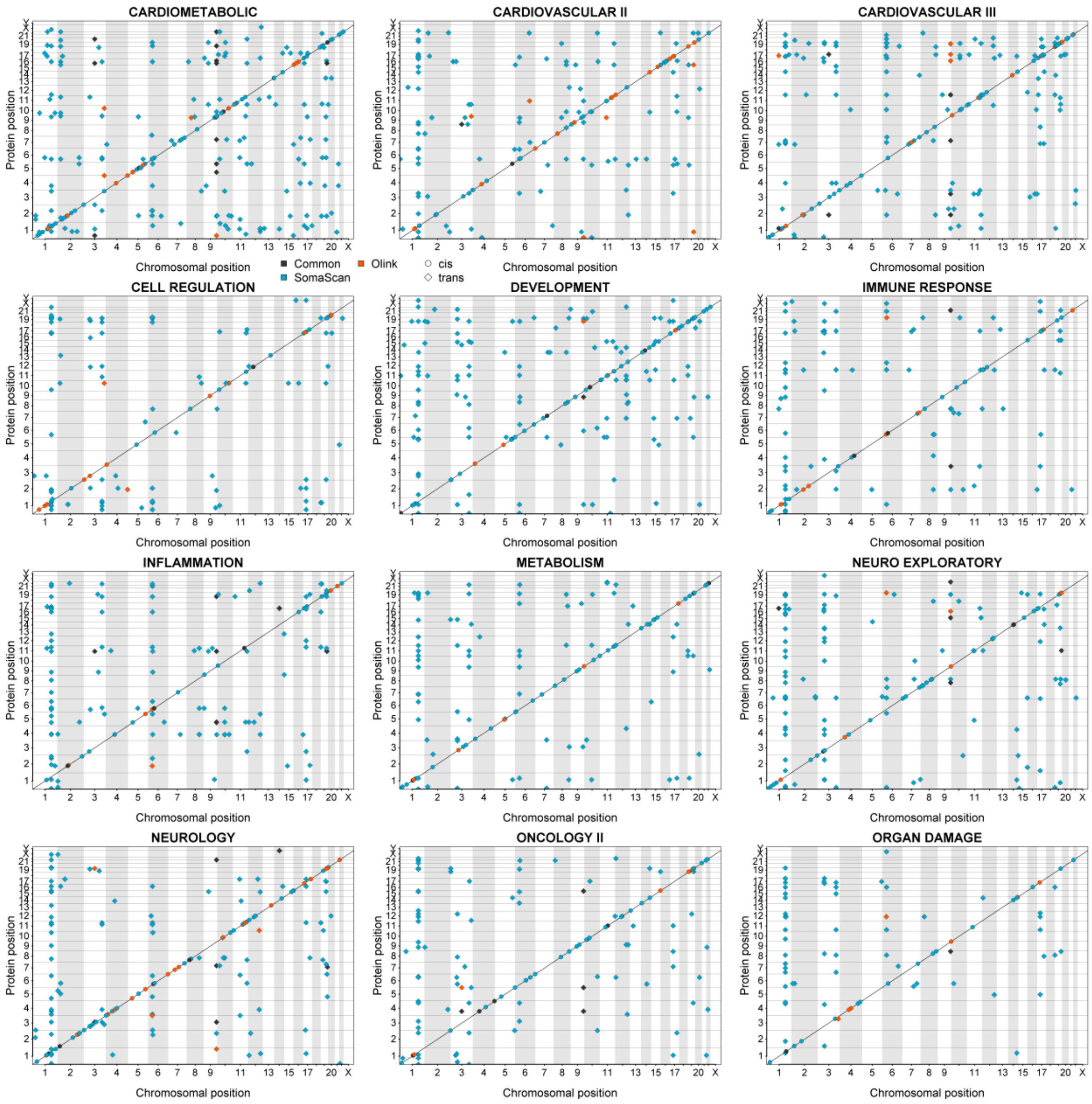

Supplementary Figure 2 Genomic location (x-axis) of single nucleotide polymorphisms (SNPs) identified to be significantly associated with at lest one out of 871 protein targets (y-axis, sorted by genomic location of the protein encoding gene) measured by SomaScan (n=10,708, $p<1.004\times10^{-11}$) or Olink (n=485, $p<4.5\times10^{-11}$). Results were grouped by Olink panel. Colours indicate whether the SNP was identified with both platforms (black), only with SomaScan (blue), or only with Olink (orange). Cirlces on the line of identify indicate SNPs within or in close proximity to the protein-encoding gene (±500kb, *cis*-protein quantitative trait loci), where as diamonds indicate SNPs in trans. Effect estimates and p-values are presented in Supplementary Data 3.

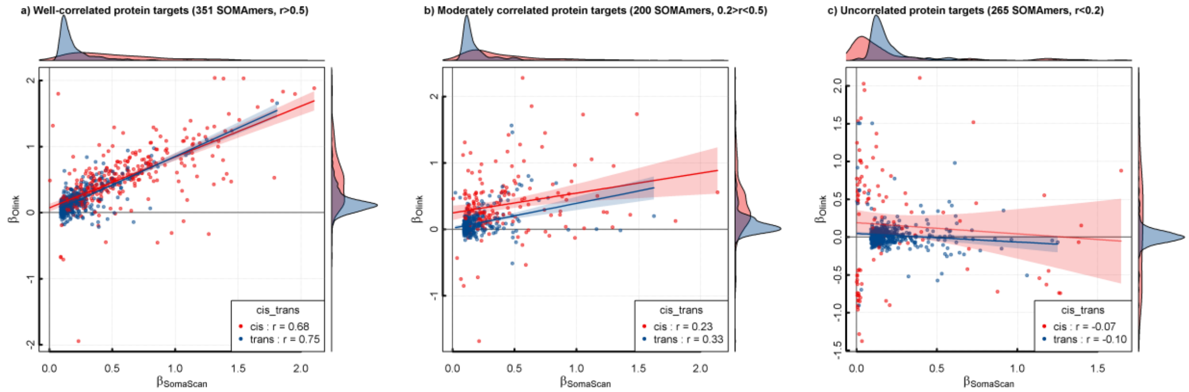

Supplementary Figure 3 Stratification of effect estimate correlations for genetic variants associated with either the SomaScan-based or Olink-based discovery. Colouring is based on the genomic location of genetic variants. Red indicates variants close to the protein encoding gene (cis, ±500kb) and blue otherwise. Estimates are presented in Supplementary Data 3.

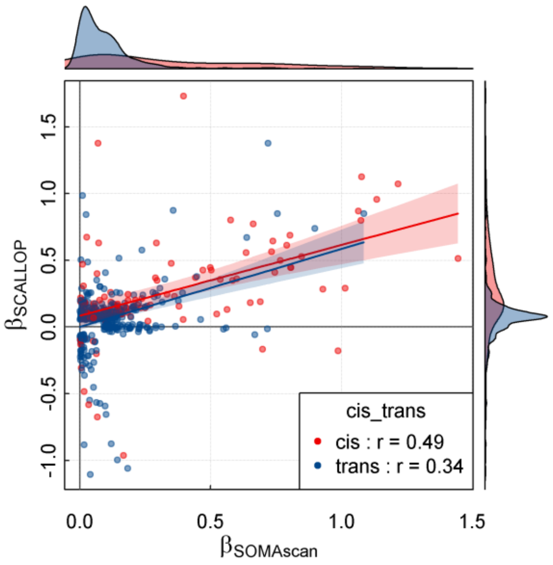

Supplementary Figure 4 Comparison of beta estimates from linear regression models across 85 corresponding SOMAmer - Olink pairs (n=77 unique protein targets) with at least one genome-wide associated genetic variant for either of the two, including 428 distinct genetic variants (R2<0.8). Genetic variants for Olink measures were derived from the most recent SCALLOP effort covering the CVD-I panel[1]. Colouring is based on the genomic location of genetic variants. Red indicates variants close to the protein encoding gene (cis, ±500kb) and blue otherwise. Estimates are presented in Supplementary Data 4.

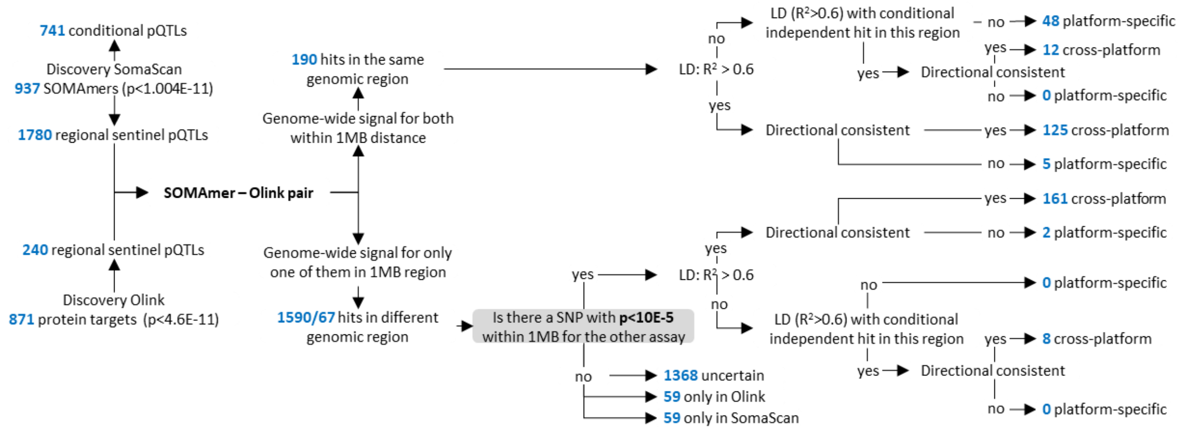

Supplementary Figure 5 Workflow to determine shared ('cross-platform') and platform-specific effects of protein-quantitative trait loci (pQTLs) between SomaScan and Olink based in the Fenland study.

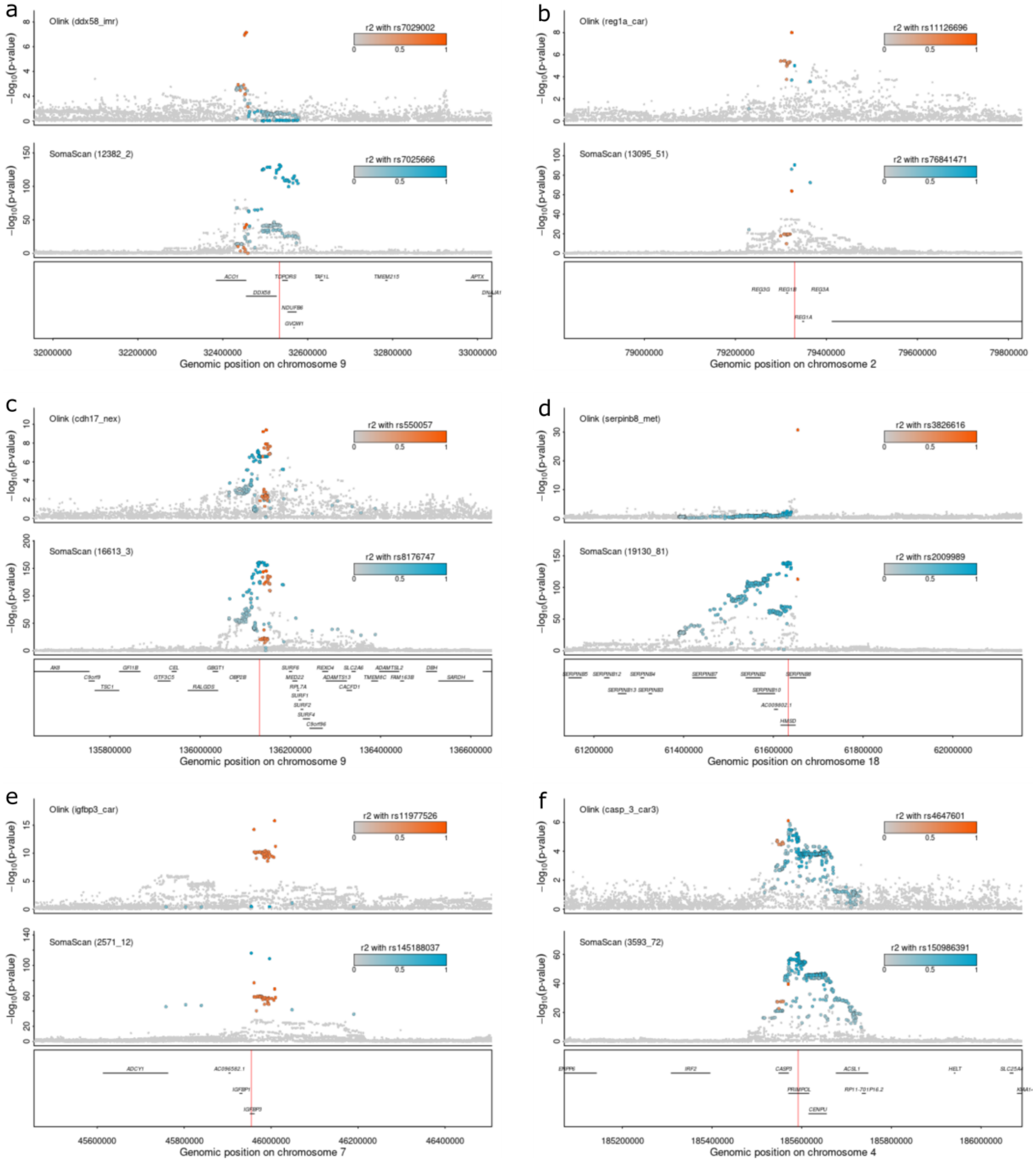

Supplementary Figure 6 Stacked regional association plots for a) DDX58, b) PSP, c) CAD17, d) SPB8, e) IGFBP-3, and f) Caspase-3 that showed evidence for a shared genetic signal that was a secondary signal for SomaScan but a lead signal for Olink. Colours indicate linkage disequilibrium (r2) with lead genetic variants for SomaScan (blue) and Olink (orange). P-values were derived from linear regression models using the protein abundances estimates by either assay as outcome and all SNPs in the region as exposure adjusting for age, sex, and genetic principal components. Source data are provided as a Source Data file.

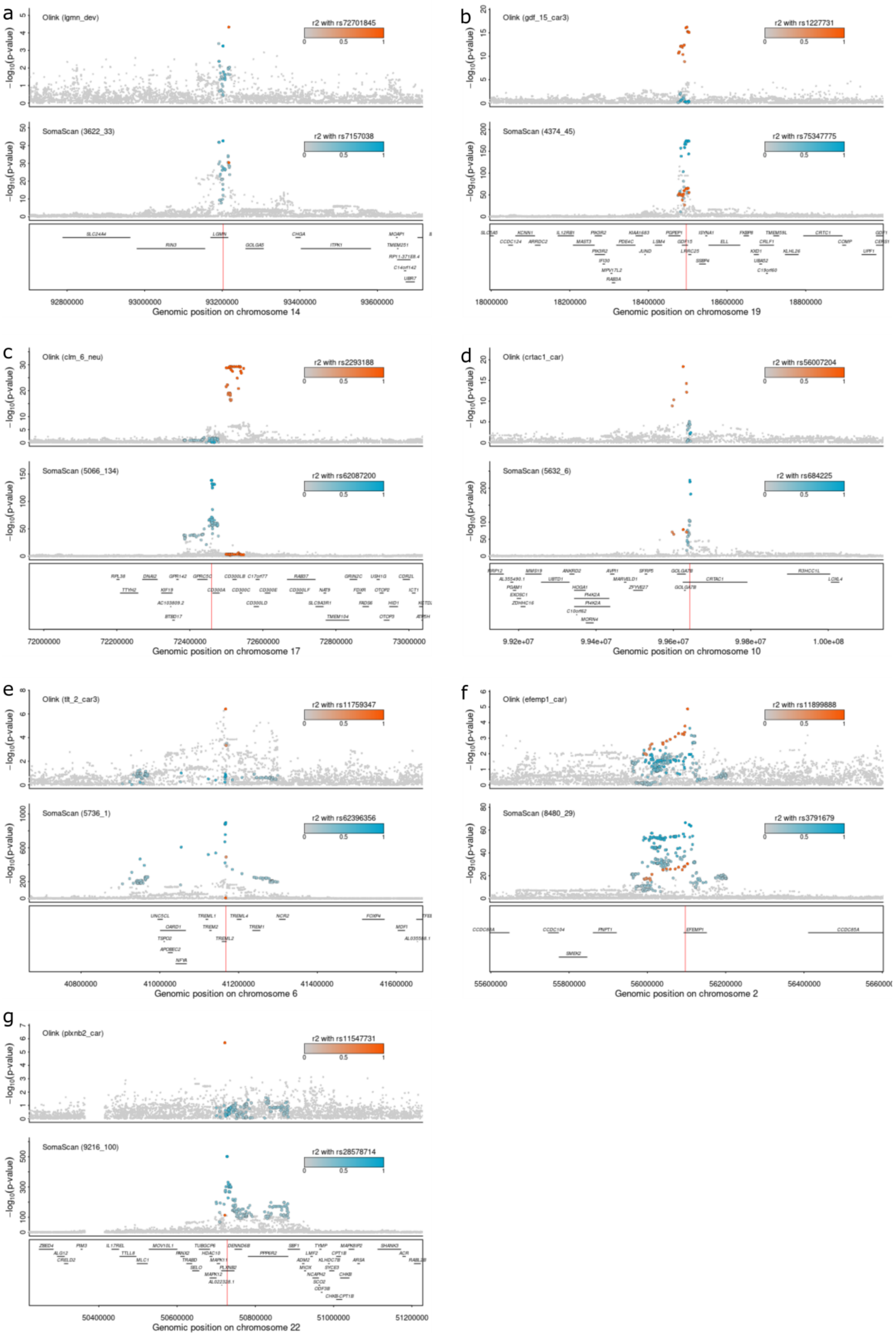

Supplementary Figure 7 Same as before but now for a) LGMN, b) MIC-1 (GDF-15), c) CLM6, d) CRAC1, e) TRML2, f) FBLN3, and g) PLXB2. P-values were derived from linear regression models using the protein

abundances estimates by either assay as outcome and all SNPs in the region as exposure adjusting for age, sex, and genetic principal components. Source data are provided as a Source Data file.

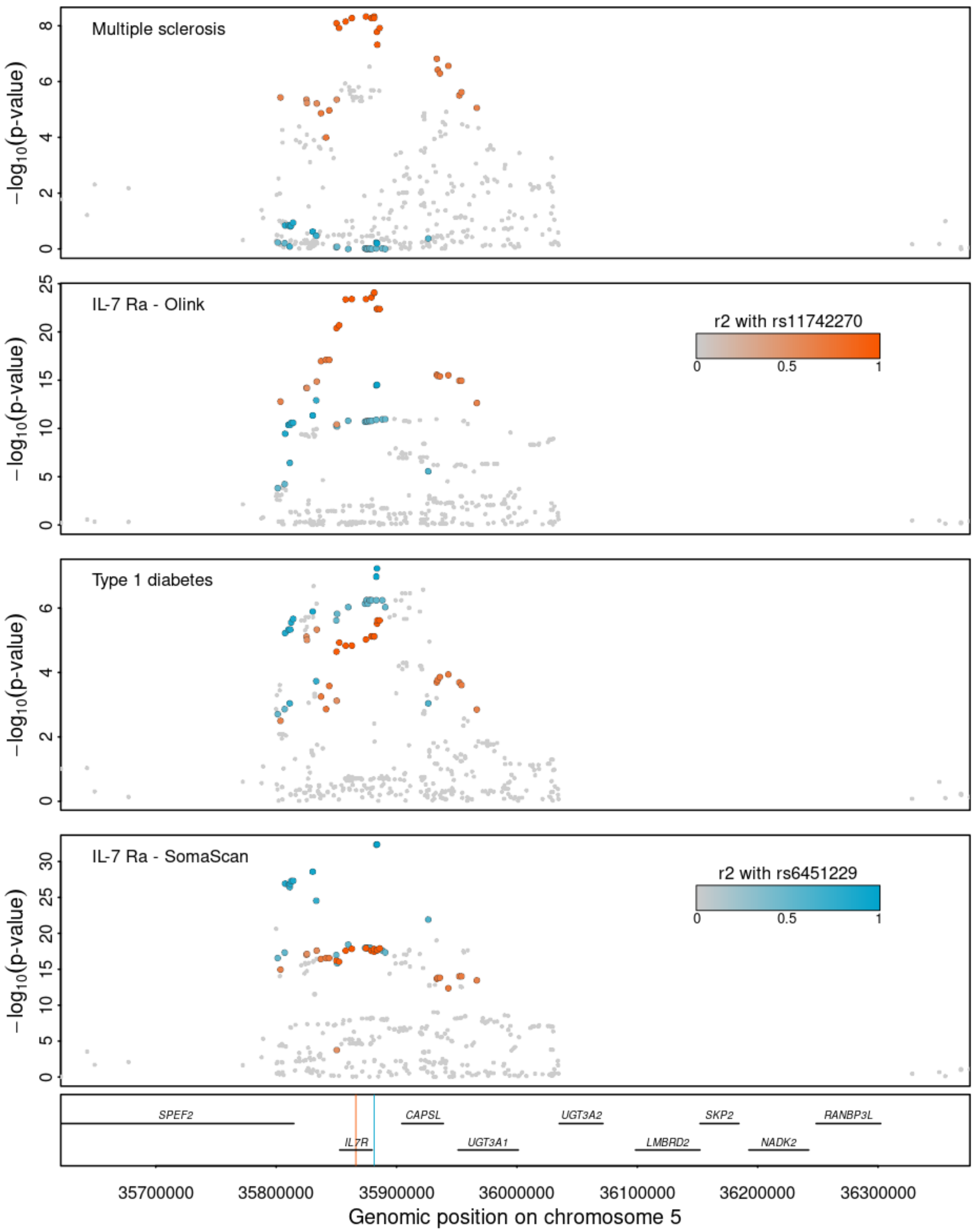

Supplementary Figure 8 Regional association plots for multiple sclerosis, type 1 diabetes, and Interleukin 7 receptor subunit alpha (IL-7 Ra) measured by Olink and SomaScan. The lead variant for each assay as well as variants in high linkage disequilibrium are highlighted by colours (blue – SomaScan, orange – Olink). Summary statistics for phenotypes were obtained from the Open GWAS database[2] or were derived from linear regression models using the protein abundances estimates by either assay as outcome and all SNPs in the region as exposure adjusting for age, sex, and genetic principal components. Source data are provided as a Source Data file.

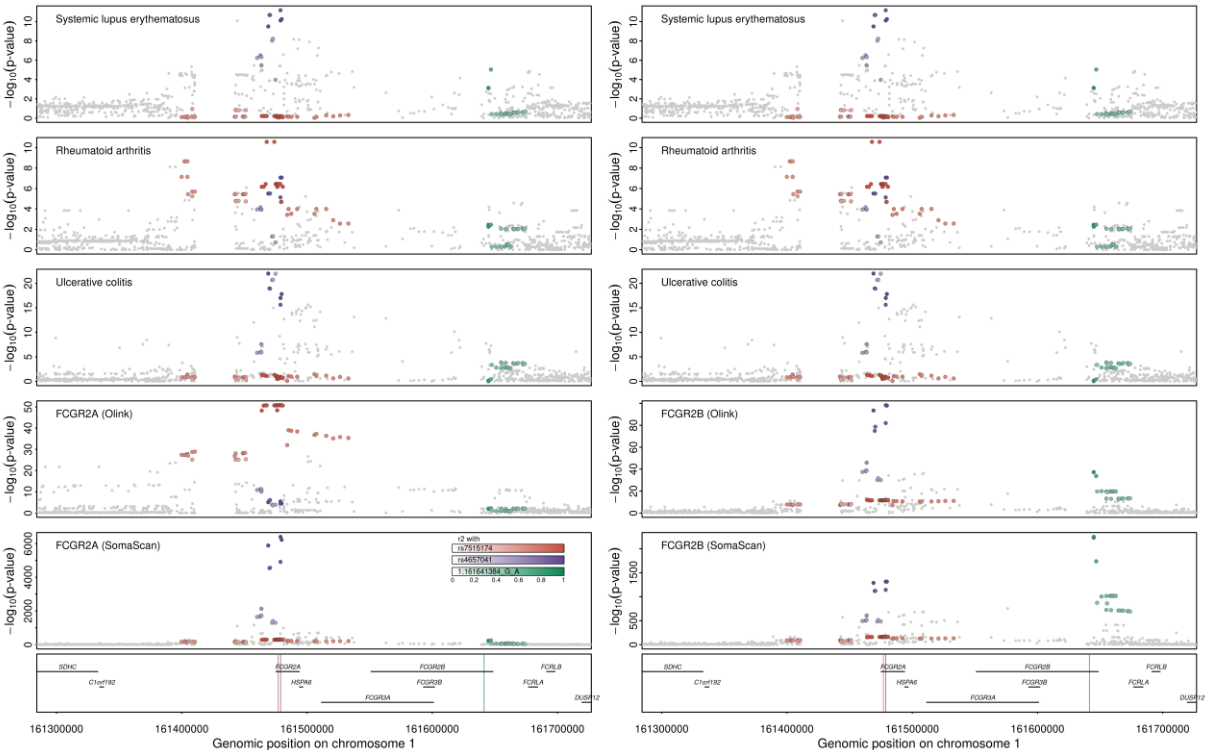

Supplementary Figure 9 Regional association plots for systemic lupus erythematosus (SLE), rheumatoid arthritis (RA), ulcerative colitis (UC), and Low-affinity immunoglobulin gamma Fc region receptor II-a (FCGR2A) and II-b (FCGR2B) as measured SomaScan and Olink. Colours indicate three lead protein quantitative trait loci and genetic variants in strong linkage disequilibrium for at least one of the protein targets. Summary statistics for SLE and RA have been obtained from the Open GWAS database[2], summary statistics for UC were obtained from de Lange et al.[3], and protein summary statistics from linear regression models using the protein abundances estimates by either assay as outcome and all SNPs in the region as exposure adjusting for age, sex, and genetic principal components. Source data are provided as a Source Data file.

.

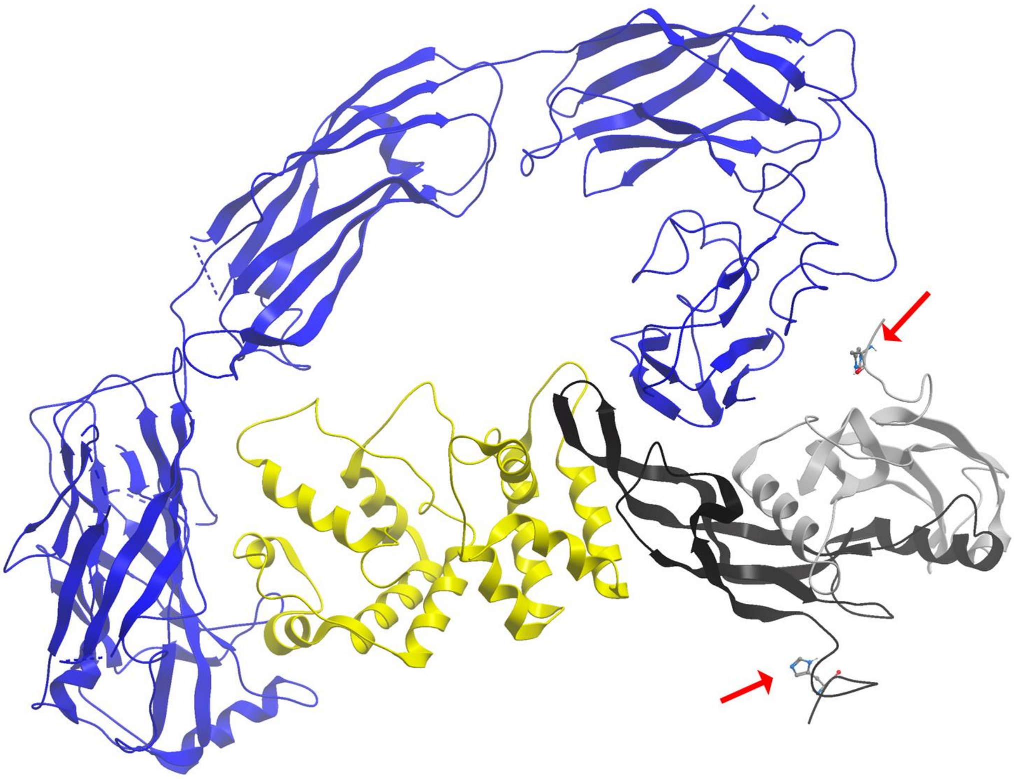

Supplementary Figure 10 Cryo-EM structure of the GDF-15 dimer (black and grey, D202 in ball and stick, arrows) bound to its receptor ectodomain composed of RET (blue) and GFRAL (yellow). The identical second half of the receptor is not shown for better clarity. Produced with PDB coordinates 6Q2J of Li et al., 2019[4].

SUPPLEMENTARY NOTE 1

Factors explaining varying correlation coefficients between measurements

We identified assay characteristics, including values below the detection limit of the assay, the affinity of the SOMAmer reagent to its protein target ('apparent Kd'), or the proportion of measurements far off from the median value ('%-outlier SomaScan/Olink' – median ±5*MAD), to be more relevant to explain varying correlation coefficients compared to any structural properties of the assayed protein targets (**Fig. 2b in main text**). We systematically tested for factors associated with the '%-outlier SomaScan' variable and identified a mix of biological (*cis*-pQTL for the SomaScan assay: inversely, p<3.1e-23) and technical factors (dilution bin: highest in the undiluted bin, p<4.7e-12; binding affinity of the SomaScan reagent: inversely, p<4.1e-10; Olink panel: highest for the inflammatory panel, p<6.5e-10) to be significantly associated. While these factors explained part of the effect, we observed many examples for which this measure identified true outlying groups consistent across both assays, including for Cripto (rs112481213, 35.6%-outlying values) and FOLR3 (rs71891516, 19.3%-outlying values) for which *cis*-pQTLs introduced bimodal distributions (**Supplementary Fig. 11**), arguing against the use a as general quality control measure to omit proteins from genetic analysis. Proteins with a transmembrane domain showed on average lower correlations compared to those without (**Fig. 2b in main text**). We suspect two possible mechanisms for this: 1) affinity reagents might target the extracellular domain of the protein and would hence measure the soluble as well as the complete protein, with important implications for the study of, e.g., inflammatory mediators activated upon cleavage form the transmembrane domain[5], or 2) the difficulty to establish correct folding of the target protein *in vivo* for affinity reagent selection.

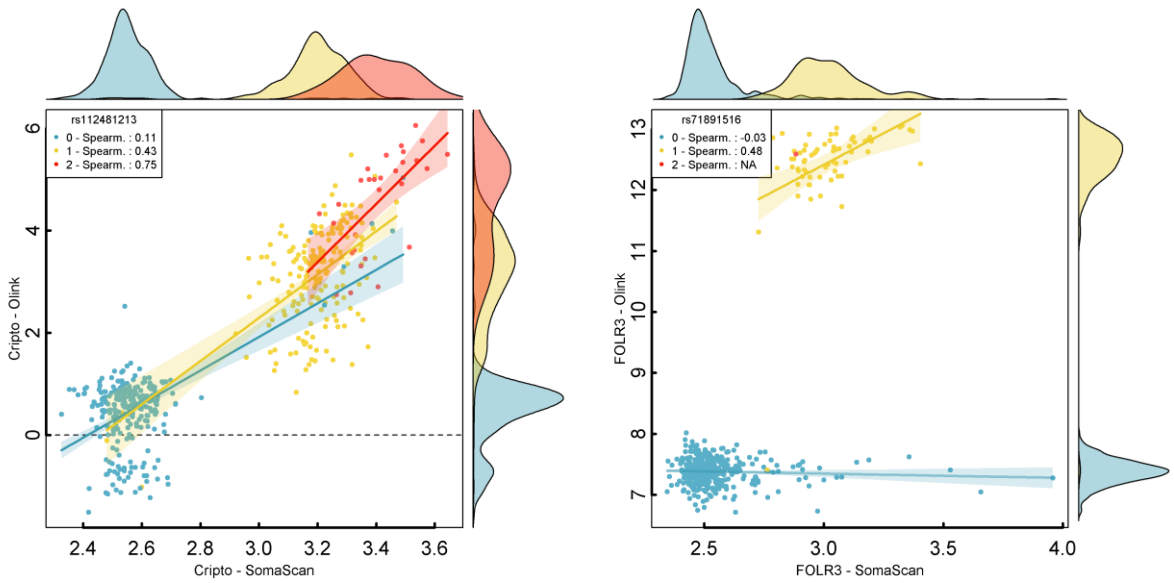

Supplementary Figure 11 Comparison of measurements for Cripto (left) and FOLR3 (right) as measured by SomaScan (x-axis) and Olink (y-axis). Points are coloured according to the genotype of the leading cis-pQTL that was shared between both platforms. The legend depicts correlation coefficients stratified by genotype. Density plots at the margins display the distributions of measurements stratified by genotype.

Non-specific trans-pQTLs in SCALLOP

To test the influence of an unbalanced design, we performed a sensitivity analysis including 307 genomic region - protein targets pairs (N=67 *cis*, N=240 *trans*, N=76 protein targets, **Supplementary Data 5**) overlapping with the SCALLOP CVD-I panel GWAS summary statistics obtained from >22,000 participants. We identified 120 (39.1%) of the pairs as cross-platform, with higher rates in *cis* (55.2%) compared to trans (34.6%) (**Supplementary Fig. 12**). The higher fraction of platform-specific pairs in *trans* (157 out of 187, 83.9%) might be best explained by two factors. Firstly, variants in *trans* might increase DNA-binding affinity of abundant circulating proteins such as complement factor H[6] (rs1061170 within *CFH*) or alter the activity of enzymes with an affinity to a large spectrum of chemical entities such as butyrylcholinesterase (rs1803274 within *BCHE* known to reduce enzymatic activity[7]) thereby possibly interfering with SOMAmer reagents. Secondly, samples taking from participants with a higher genetic susceptibility to (white) blood cell counts are possibly more prone to analytical artefacts during sample preparation, such as cell lysis and subsequent spill over of proteins into the plasma. The pleiotropic *trans*-pQTL rs3443671 within *NLRP12* might be such an example, since neither we or other SomaScan-based discovery efforts[8] were able to replicate this pleiotropic association, which is a known blood cell locus[9]. Out of the 140 platform-specific *trans*-pQTLs, 31 and 25, respectively, were likely attributable to those reasons.

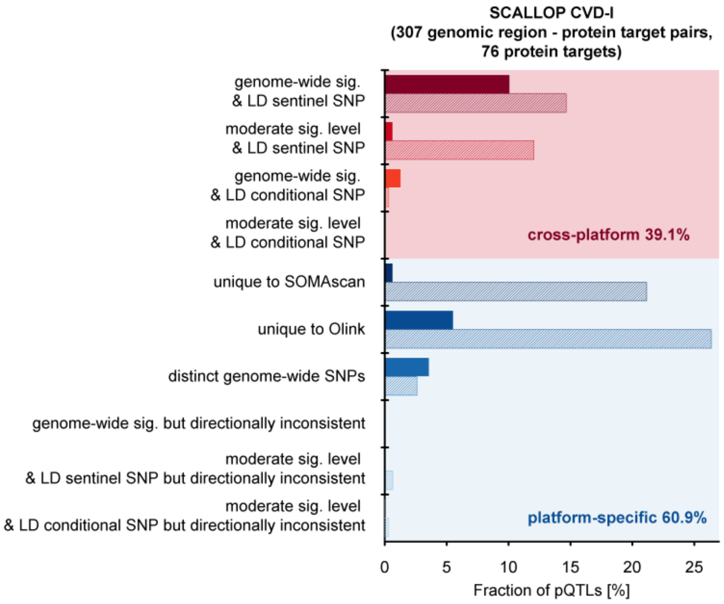

Supplementary Figure 12 Summary of platform agreement for 307 genomic region – protein target associations with sufficient power in the Fenland SomaScan study and the SCALLOP CVD-I consortium. Protein quantitative trait loci (pQTL) close to the protein encoding gene (±500kb) are depicted as filled bars, whereas pQTLs outside this region are depicted by shaded bars *(trans*-pQTL).

Genetic variants account for measurement differences

We identified multiple *trans*-pQTLs that changed the correlation between overlapping protein measures. For example, variants mapping to genes encoding for ubiquitously expressed glycosyltransferases may act through altered glycosylation of protein targets affecting the accessibility for affinity reagents. We observed two such examples, namely rs281379 (associated with TECK and in LD, r^2=0.83, with a missense variant in *FUT2*) and rs779860630 (associated with SARP-2 and located in the intron of *ABO*) mapping to genes encoding glycosyltransferases. Apart from altered affinity to binding reagents, increased glycosylation of protein targets has been shown to increase stability of the 3-dimensional structure of proteins[10] and diminished glycosylation might hence reduce the amount of correctly folded proteins circulating, which is a prerequisite of SOMAmer reagents to correctly bind. Another possibility is a higher affinity for RNA- or DNA-binding of the gene product conferred by the genetic variant. We observed rs9501393 (MAF=13.5%) modulating the correlation coefficient of Endothelin-converting enzyme 1 (**Fig. 3d in main text**). rs9501393 is in strong LD (r^2=0.94) with a missense variant of uncertain significance in *SKIV2L* (rs449643, p.A1071V) encoding an RNA helicase, a protein with high affinity to bind to RNA or single-stranded DNA oligomers.

We identified factors that influenced measurement differences at the individual participant data level, considering pQTLs as well as phenotypic measures that could have an impact on protein

abundances, namely age, sex, body mass index (BMI), estimated glomerular filtration (eGFR; calculated from serum creatinine, age, and sex), and plasma alanine transaminase activities (ALT). The combination of all factors explained a median amount of 5.6% (IQR: 3.5% - 9.2%) of the differences in measurements reaching values of up to 69.4% for YKL-40 (**Supplementary Fig. 13**). For 211 (23%) out of 814 protein targets with at least one pQTL, the pQTL accounted for most of the explained variance (median: 1.0%, IQR: 0.2% - 3.4%), including 85 protein targets with >10%. The strong contribution of certain genetic variants aligns with the results for platform-specific *cis*- and *trans*-pQTLs outlined above.

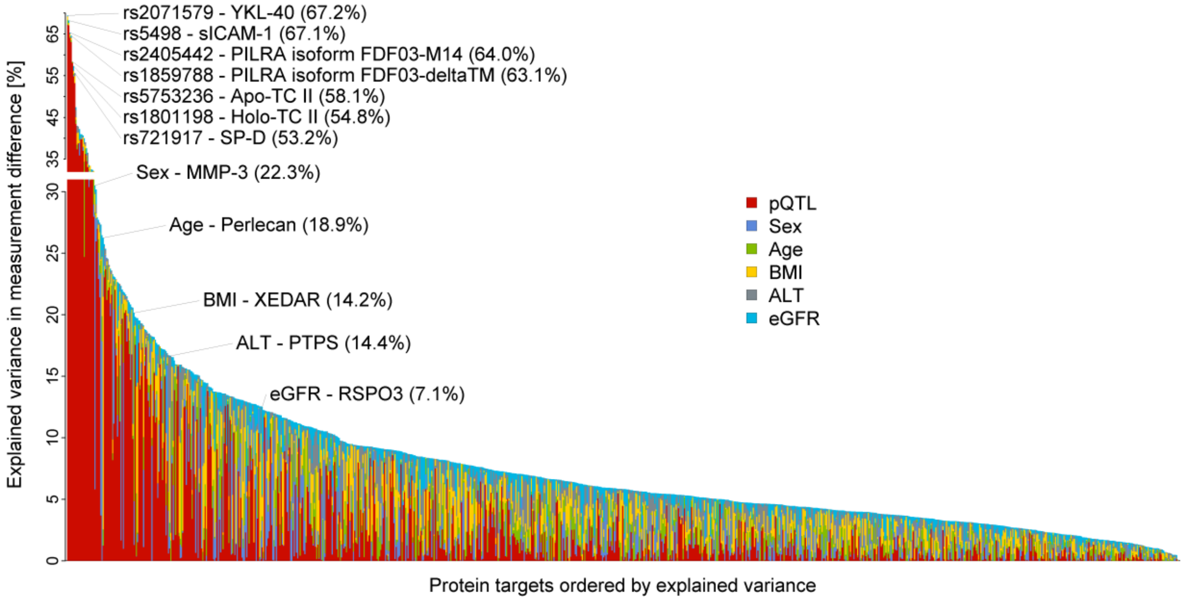

Supplementary Figure 13 Protein targets ordered by the amount of variance explained in the differences between measurements based on SomaScan and Olink. Contribution of protein quantitative trait loci (pQTL), age, sex, body mass index (BMI), plasma alanine aminotransferase activities (ALT), and estimated glomerular filtration rate (eGFR) are given in colours. Selected protein targets are annotated. Source data are provided as a Source Data file.

SUPPLEMENTARY REFERENCES

1. Folkersen, L. *et al.* Genomic and drug target evaluation of 90 cardiovascular proteins in 30,931 individuals. *Nat. Metab.* **2**, 1135–1148 (2020).

2. Elsworth, B. *et al.* The MRC IEU OpenGWAS data infrastructure. *bioRxiv* 2020.08.10.244293 (2020) doi:10.1101/2020.08.10.244293.

3. De Lange, K. M. *et al.* Genome-wide association study implicates immune activation of multiple integrin genes in inflammatory bowel disease. *Nat. Genet.* **49**, 256–261 (2017).

4. Li, J. *et al.* Cryo-EM analyses reveal the common mechanism and diversification in the activation of RET by different ligands. *Elife* **8**, 1–26 (2019).

5. Lichtenthaler, S. F., Lemberg, M. K. & Fluhrer, R. Proteolytic ectodomain shedding of membrane proteins in mammals—hardware, concepts, and recent developments. *EMBO J.* **37**, (2018).

6. Sjöberg, A. P. *et al.* The factor H variant associated with age-related macular degeneration (His-384) and the non-disease-associated form bind differentially to C-reactive protein, fibromodulin, DNA, and necrotic cells. *J. Biol. Chem.* **282**, 10894–900 (2007).

7. Sohail, I. & Rashid, S. Molecular dynamics and regulation of butyrylcholinesterase cholinergic activity by RNA binding proteins. *CNS Neurol. Disord. Drug Targets* **13**, 1366–1377 (2014).

8. Gudjonsson, A. *et al.* A genome-wide association study of serum proteins reveals shared loci with common diseases. *bioRxiv* 2021.07.02.450858 (2021) doi:10.1101/2021.07.02.450858.

9. Astle, W. J. *et al.* The Allelic Landscape of Human Blood Cell Trait Variation and Links to Common Complex Disease. *Cell* **167**, 1415-1429.e19 (2016).

10. Shental-Bechor, D. & Levy, Y. Effect of glycosylation on protein folding: A close look at thermodynamic stabilization. *Proc. Natl. Acad. Sci. U. S. A.* **105**, 8256–8261 (2008).
