# Supplementary material for: Synergistic insights into human health from aptamer- and antibody-based proteomic profiling
Source: Nat Commun. 2021 Nov 24;12:6822. doi: 10.1038/s41467-021-27164-0 (PMC8613205; doi:10.1038/s41467-021-27164-0)
Supplement: Supplementary file 3 — Description of Additional Supplementary Files [file 41467_2021_27164_MOESM3_ESM.docx]

**Description of Additional Supplementary Files**

**File Name:** Supplementary Data 1

**Description:** Baseline characteristics of Fenland participants

**File Name:** Supplementary Data 2

**Description:** Summary of overlapping protein targets between the SomaScan v4 assay and 12 Olink panels

**File Name:** Supplementary Data 3

**Description:** Reciprocal look-up of pQTLs between the SomaScan-based and Olink-based genetic discovery

**File Name:** Supplementary Data 4

**Description:** Reciprocal look-up of pQTLs between the SomaScan-based and SCALLOP-based genetic discovery

**File Name:** Supplementary Data 5

**Description:** Consistency of genomic regions with sufficient power to compare results from the SomaScan-based and Olink-based discovery

**File Name:** Supplementary Data 6

**Description:** Consistency of genomic regions with sufficient power to compare results from the SomaScan-based and SCALLOP-based discovery

**File Name:** Supplementary Data 7

**Description:** Meta-data used to predict likelihood of consistent pQTLs in the Fenland dataset

**File Name:** Supplementary Data 8

**Description:** Meta-data used to predict likelihood of consistent pQTLs in the SCALLOP dataset

**File Name:** Supplementary Data 9

**Description:** Results from logistic regression models to test for the likelihood of consistency

**File Name:** Supplementary Data 10

**Description:** Protein characteristics and their effect on aptamer- and antibody-based measurements of protein targets

**File Name:** Supplementary Data 11

**Description:** Results from phenome-wide colocalisation analysis for protein encoding regions
