# Supplementary material for: Synergistic insights into human health from aptamer- and antibody-based proteomic profiling
Source: Nat Commun. 2021 Nov 24;12:6822. doi: 10.1038/s41467-021-27164-0 (PMC8613205; doi:10.1038/s41467-021-27164-0)
Supplement: Supplementary file 15 — Reporting Summary [file 41467_2021_27164_MOESM15_ESM.pdf]

nature portfolio

Corresponding author(s): Claudia Langenberg

Last updated by author(s): Oct 27, 2021

Reporting Summary

Nature Portfolio wishes to improve the reproducibility of the work that we publish. This form provides structure for consistency and transparency in reporting. For further information on Nature Portfolio policies, see our Editorial Policies and the Editorial Policy Checklist.

Statistics

For all statistical analyses, confirm that the following items are present in the figure legend, table legend, main text, or Methods section.

n/a	Confirmed	
☐	☒	The exact sample size (n) for each experimental group/condition, given as a discrete number and unit of measurement
☐	☒	A statement on whether measurements were taken from distinct samples or whether the same sample was measured repeatedly
☐	☒	The statistical test(s) used AND whether they are one- or two-sided<br>*Only common tests should be described solely by name; describe more complex techniques in the Methods section.*
☐	☒	A description of all covariates tested
☐	☒	A description of any assumptions or corrections, such as tests of normality and adjustment for multiple comparisons
☐	☒	A full description of the statistical parameters including central tendency (e.g. means) or other basic estimates (e.g. regression coefficient) AND variation (e.g. standard deviation) or associated estimates of uncertainty (e.g. confidence intervals)
☐	☒	For null hypothesis testing, the test statistic (e.g. F, t, r) with confidence intervals, effect sizes, degrees of freedom and P value noted<br>*Give P values as exact values whenever suitable.*
☐	☒	For Bayesian analysis, information on the choice of priors and Markov chain Monte Carlo settings
☒	☐	For hierarchical and complex designs, identification of the appropriate level for tests and full reporting of outcomes
☐	☒	Estimates of effect sizes (e.g. Cohen's d, Pearson's r), indicating how they were calculated

Our web collection on statistics for biologists contains articles on many of the points above.

Software and code

Policy information about availability of computer code

Data collection	N/A
Data analysis	IMPUTE4, BGENIE (v1.3), PLINK v2.0, R v3.6.0 (packages: coloc v4.0.4, ieugwasr v0.1.5, variancePartition v1.14.1, igraph v1.2.6), METAL (version from 2011-03-25), GCTA-cojo v.1.90, STATA v14, VEP v98.3. Details of specific software and references can be found within text in the relevant Methods sections.

For manuscripts utilizing custom algorithms or software that are central to the research but not yet described in published literature, software must be made available to editors and reviewers. We strongly encourage code deposition in a community repository (e.g. GitHub). See the Nature Portfolio guidelines for submitting code & software for further information.

Data

Policy information about availability of data

All manuscripts must include a data availability statement. This statement should provide the following information, where applicable:

- Accession codes, unique identifiers, or web links for publicly available datasets
- A description of any restrictions on data availability
- For clinical datasets or third party data, please ensure that the statement adheres to our policy

Information about the Fenland cohort is available at the study website (https://www.mrc-epid.cam.ac.uk/research/studies/fenland/information-for-researchers/), which includes a link to the MRC Epidemiology Unit metadata access portal (https://epi-meta.mrc-epid.cam.ac.uk/). To comply with the consent given by Fenland participants, data access is granted to bona fide researchers through an application process that typically takes no more than 4-6 weeks. Data will either be shared through an institutional data sharing agreement or arrangements will be made for analyses to be conducted remotely without the necessity for data transfer. Publicly available summary statistics for look-up and colocalisation of pQTLs were obtained from https://gwas.mrcieu.ac.uk/ and https://www.ebi.ac.uk/gwas/. We

obtained genome-wide summary statistics for 90 protein targets from Folkersen et al., which are also available from the GWAS catalog (GCST90011994-GCST90012083).

Field-specific reporting

Please select the one below that is the best fit for your research. If you are not sure, read the appropriate sections before making your selection.

☒ Life sciences ☐ Behavioural & social sciences ☐ Ecological, evolutionary & environmental sciences

For a reference copy of the document with all sections, see nature.com/documents/nr-reporting-summary-flat.pdf

Life sciences study design

All studies must disclose on these points even when the disclosure is negative.

Sample size	We used individual level data from 10,708 participants of the Fenland cohort with available genotype and proteomic data.
Data exclusions	We excluded 1727 participants with missing genotype data, before analysing the data.
Replication	A key aim of the study was to identify factors associated with the reproducibility of genetic findings across two distinct proteomic techniques in up to 10,708 participants and we seeked external comparison including up to 22,000 samples for 90 proteins as published by Folkersen et al. 2021 Nature Metabolism. We replicated a total of 306 (63.9%) and 120 (39.1%) genomic region - protein target associations in our internal and external data sets, respectively. Reasons for non-replicating results included differences in the mode of measurement of protein targets between techniques and study-specific blood sampling artefacts as described in detail in the main text.
Randomization	All genetic associations were controlled for age, sex, test site, and population stratification.
Blinding	Blinding does not apply as no case/control analysis have been done, but rather analysis of continuous outcomes.

Reporting for specific materials, systems and methods

We require information from authors about some types of materials, experimental systems and methods used in many studies. Here, indicate whether each material, system or method listed is relevant to your study. If you are not sure if a list item applies to your research, read the appropriate section before selecting a response.

Materials & experimental systems

n/a	Involved in the study	
☒	☐	Antibodies
☒	☐	Eukaryotic cell lines
☒	☐	Palaeontology and archaeology
☒	☐	Animals and other organisms
☐	☒	Human research participants
☒	☐	Clinical data
☒	☐	Dual use research of concern

Methods

n/a	Involved in the study	
☒	☐	ChIP-seq
☒	☐	Flow cytometry
☒	☐	MRI-based neuroimaging

Human research participants

Policy information about studies involving human research participants

Population characteristics	Fenland participants were on average 48.6 years old (standard deviation: 7.5 years) and 53.4% were female. A maximum set of 10,708 participants with available genotypes have been included in the analysis.
Recruitment	Fenland is a population-based cohort study of 12,435 participants without diabetes born between 1950 and 1975. Participants were recruited from general practice surgeries in the Cambridgeshire region of the UK and underwent detailed phenotyping at a baseline visit between 2005 and 2015. Exclusion criteria were clinically diagnosed diabetes mellitus, inability to walk unaided, terminal illness (life expectancy of <1 year at the time of recruitment), clinically diagnosed psychotic disorder, pregnancy, or lactation.
Ethics oversight	The study was approved by the Cambridge Local Research Ethics Committee (NRES Committee – East of England Cambridge Central, ref. 04/Q0108/19) and all participants provided written informed consent. The consent covered measurements made from blood samples as well as extends beyond the baseline examination.

Note that full information on the approval of the study protocol must also be provided in the manuscript.
